# Roles of the Cholinergic and Adrenergic Systems in Vagus Nerve Stimulation for the Recovery of Motor Function in Patients with Stroke: Opportunities for Novel Treatments and Rehabilitation

**DOI:** 10.3390/ijms27114701

**Published:** 2026-05-23

**Authors:** Auwal Abdullahi, Thomson W. L. Wong, Shamay S. M. Ng

**Affiliations:** 1 Department of Physiotherapy, Bayero University, Kano 700006, Nigeria; aabdullahi.pth@buk.edu.ng; 2 Department of Physiotherapy, Federal University Wukari, Wukari 670102, Nigeria; 3 Department of Physiotherapy, Federal Medical Center Nguru, Nguru 630261, Nigeria; 4 Department of Rehabilitation Sciences, The Hong Kong Polytechnic University, Hong Kong, China; thomson.wong@polyu.edu.hk

**Keywords:** stroke, vagus nerve stimulation, cholinergic system, adrenergic system, motor recovery

## Abstract

Impairment in blood supply to the brain deprives its cells of the much-needed nutrients and molecules such as oxygen and glucose necessary for its development, growth and survival. This will set up a host of pathological processes such as impaired homeostasis, energy failure, excitotoxicity, oxidative stress, impaired protein synthesis, inflammation, cytokine-mediated toxicity and impairment of blood–brain barrier. These pathological processes will result in the damage or death of the cells depending on the extent of the deprivation. Similarly, they will impair synthesis of acetylcholine (Ach) and norepinephrine (NE), which are important neurotransmitters in the cholinergic and adrenergic systems responsible for cellular communication and functions. Thus, interventions to help arrest and/or modulate the initial and subsequent pathological states and help recover the functions of the brain are needed. One of such interventions is vagus nerve stimulation, which helps activate the cholinergic and the adrenergic systems via projections of the afferent fibers of the vagus nerve to the nucleus of the solitary tract (NTS). Activation of the cholinergic and the adrenergic systems results in reduction in pro-inflammatory factors such as tumor necrosis α, increase in pro-angiogenic factors and increase in firing of adrenergic neurons in the central nervous system (CNS).

## 1. Expert Opinion

Stroke is caused by inadequate supply of blood to the neuronal cells in the brain. Normally, the brain receives adequate blood that carries oxygen and other essential nutrients necessary for the growth, maturation and functions of its cells. However, when supply of blood to the brain cells becomes inadequate, a host of pathological events such as abnormal homeostasis, energy failure, excitotoxicity, oxidative stress, impaired protein synthesis, inflammation, cytokine-mediated toxicity, complement activation, impairment of blood–brain barrier, acidosis, increased intracellular calcium levels, free-radical-mediated toxicity, activation of glial cells, and infiltration of leukocytes occur. These pathological events will in turn cause damage or death of the neuronal cells in the brain, depending on the extent or the length of the pathological events. Similarly, damage or death of the neuronal cells will impair the cholinergic and the adrenergic systems by disrupting synthesis of acetylcholine (Ach) and norepinephrine (NE), respectively, which are important neurotransmitters responsible for cellular communication and functions.

Under normal circumstance, the cholinergic system is involved in various physiological processes in the brain such as learning and memory, attention, sleep, arousal and regulation of homeostasis. In particular its role in the physiological processes of learning and memory plays a major role in the recovery of functions of the central nervous system CNS following an injury such as stroke. It is involved in these two neurophysiological processes by modulating neural activity via acetylcholine receptors. Similarly, the adrenergic system comprises epinephrine, norepinephrine (NE), adrenergic receptors (ARs), tyrosine hydroxylase, dopa carboxylase and dopamine β-hydroxylase. In the brain, the Locus Coeruleus (LC) is the main noradrenergic nucleus and the main site for the synthesis of norepinephrine. The Locus Coeruleus (LC)–norephinephrine (NE) system consists of neurons that project to different brain regions and supplies NE to the cortex, hippocampus, striatum, amygdala, cerebellum, basal forebrain, and the hypothalamus. This is made possible because, in these regions of the brain, adrenergic receptors are widely distributed. Thus, the adrenergic system together with NE are involved in the process of memory and learning and play important roles in motor control, attention and cognitive functions. Consequently, due to the numerous diffuse cortical connections of the vagus nerve, activation of the adrenergic system helps recover motor function in conjunction with cholinergic system.

Activation of the cholinergic and the adrenergic systems occurs via the projections of the afferent fibers of the vagus nerve to the nucleus of the solitary tract (NTS). This activation results in reduction in pro-inflammatory factors such as tumor necrosis α, increase in pro-angiogenic factors that characterize stroke pathology, and increase the firing of adrenergic neurons in the CNS. Thus, in our opinion, interventions that will target and/or modulate the Ach, NE and their receptors are needed to help patients with stroke recover functions.

## 2. Introduction

Stroke is a neurological disease caused by impairment in the supply of blood to the brain as a result of reduced caliber or occlusion and/or rupture of the blood vessels supplying the brain [1,2,3,4,5]. When the blood vessels supplying the brain get occluded or ruptured, supply of essential molecules and nutrients important for the survival of the brain cells gets impaired as well, leading to a cascade of pathological processes that result in neuronal cell damage and/or death [2,6,7]. These cascades of pathological processes that occur during stroke include impaired homeostasis, energy failure, excitotoxicity, oxidative stress, impaired protein synthesis, inflammation, cytokine-mediated toxicity, complement activation, impairment of blood–brain barrier, acidosis, increased intracellular calcium levels, free-radical-mediated toxicity, activation of glial cells, and infiltration of leukocytes and programed cell death [8,9,10].

**Figure 1:**
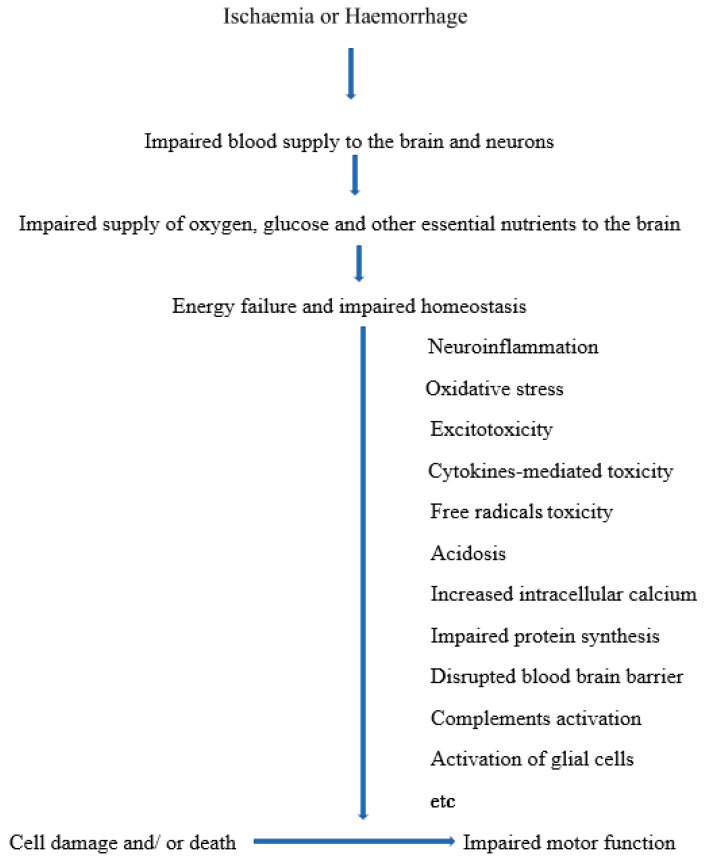
Details of pathological processes in stroke that result in impairment in motor function.

The above pathological processes especially the energy failure, impaired homeostasis and inhibition of protein synthesis, in turn, impair synthesis of acetylcholine (Ach) and norepinephrine (NE), which are important neurotransmitters in the cholinergic and adrenergic systems responsible for cellular communication and functions [11,12] (see Figure 1 for the details of pathological processes in stroke that result in impairment in motor function). Consequently, the survival of the neuronal cells is put in danger and, as such, they may get damaged or die and subsequently injure or lead to the death of the neighboring healthy neuronal cells [2]. Damage or death of these cells will lead to the impairment in functions of the brain, which includes motor, sensory/perceptual, cognitive and autonomic functions that subsequently result in disability in carrying out activities of daily living (ADL) [13,14]. Thus, interventions to help arrest and/or modulate the initial and subsequent pathological states and help recover the functions of the brain are needed. One of such interventions is vagus nerve stimulation (VNS) [15,16,17,18,19,20,21,22].

However, despite the emerging evidence on the role of VNS in motor recovery following stroke, its use in practice seems to be limited. The aim of this review is to therefore discuss the roles played by the cholinergic and the adrenergic systems in VNS for the rehabilitation of motor function in patients with stroke. This may help provide more confidence for its use in practice.

## 3. The Vagus Nerve and Vagus Nerve Stimulation (VNS)

Vagus nerve stimulation (VNS) is believed to have first been used by Dr James Corning in the 1880s [23,24]. Dr Corning made an attempt to electrically stimulate the vagus nerve in order to treat seizures [24]. It was further advanced by Jacob Zabara in the 1980s, when he developed an implantable neurocybernetic prosthesis [25,26]. This was followed in 1997 by approval from the US Food and Drug Administration (FDA) to use invasive VNS (iVNS) to treat medically refractory epilepsy [27]. However, considerations primarily because of cost of the iVNS paved way for the introduction of non-invasive VNS [28,29]. Subsequently, use of iVNS and non-invasive VNS was applied for patients with stroke in order to improve motor function [17,30].

Vagus nerve stimulation (VNS) is used to stimulate the vagus nerve [30,31]. The vagus nerve is the cranial nerve number 10, which is the longest cranial nerve in the body [32]. It extends from the brainstem to as far down as the colon and, in doing so, it traverses and influences many structures that are vital to human body functions [33].

In its relationship with the function of many structures in the body as stated above, the nerve serves both afferent and efferent motor and sensory functions [30,34,35]. The afferent function is carried out by the afferent fibers arising from the nodose ganglion and projecting largely to the nucleus of the solitary tract (NTS) [36,37,38]. Similarly, the efferent is carried out by the efferent fibers arising from the NTS to other brain regions through direct projections to the parabrachial nucleus (PBN), the cerebellum, the raphae, the periaqueductal gray (PAG), and the locus coeruleus, as well as ascending secondary projections to limbic, paralimbic, and cortical regions [39]. In particular, the projection of the afferent fibers to the NTS helps to rapidly activate the cholinergic and the noradrenergic systems [40,41]; and what is interesting is that this neurological mechanism may be activated or enhanced using VNS to help with recovery of motor function following stroke.

The VNS is a neurophysiological technique of stimulating the vagus nerve directly (invasive) by means of an implant or indirectly (non-invasive) by means of stimulating the auricular, cervical and diaphragmatic branches of the nerve [41]. For the invasive technique, the nerve is identified in the midline at the level of cricoid cartilage following an incision, and then a stimulation lead is wrapped around it [16,17,20]. The lead is connected to a pulse generator device stationed under the skin in the pectoral region [20]. The device is activated using a wireline switch or a push button on the device. Similarly, the non-invasive VNS is carried out using a transcutaneous electrical stimulator with an electrode placed over the left external acoustic meatus at the inner side of the tragus or modified dot-like electrodes that are fitted to the cymba conchae [15,18,19]. However, it should be noted that, although the invasive technique is said to produce a better effect on motor function than the invasive type, it also causes more adverse events [30].

The parameters used for the stimulation vary. In some studies, the stimulation parameters used are 0.8 mA, constant current, and charge balanced pulses (100 μs pulse width; 30 Hz frequency) [16,17,20]. In other studies, the stimulation parameters used are 600 pulses (intratrain pulse frequency = 20 Hz; pulse duration = 0:3 ms) [15,18,21]. Similarly, single 500 ms bursts with a frequency of 30 Hz and a pulse width of 0.3 ms are also used as stimulation parameters [19]. When 0.8 mA, constant current and charge balanced pulses (100 μs pulse width; 30 Hz frequency) were used as stimulation parameters; a reduction in infarct volume was observed [16]. This reduction could be because of activation of the cholinergic system that resulted in reduction in pro-inflammatory factors such as tumor necrosis α (TNF-α) [42,43,44,45]. In addition, projection of the afferent fibers of the vagus nerve to the NTS has been said to help activate the cholinergic and the noradrenergic systems, which are important for various functions of the central nervous system (CNS) [39,40].

However, it should be noted that the above studies [15,16,17,18,19,20,21] have different characteristics. For instance, in one of the studies [16], only patients with ischemic stroke were used; while, in another study [15], patients with either ischemic or hemorrhagic stroke were used. Similarly, while a study [17] used patients with chronic stroke, another study [18] used patients with sub-acute stroke. In addition, some of the studies [15,18] are pilot controlled trials with low sample sizes. Furthermore, different modes of administration of VNS were used in the studies. In particular, four of the studies [15,18,19,21] used iVNS. Therefore, clear information on the intensity of VNS to use or the characteristics of patients that require what is still lacking, suggesting that further studies are needed.

In addition, selection of the parameters for the VNS is critical to the effects it produces. High-frequency stimulation of >20 Hz resulted in a more robust engagement of the locus coeruleus, with a corresponding sustained release of norepinephrine release, which is essential for long-term potentiation-like plasticity in motor circuits [46]. In contrast, a continuous low-frequency stimulation resulted in a preferential activation of vagal afferents that promote anti-inflammatory cascades without sufficiently driving noradrenergic-mediated plasticity [47]. This distinction has significant clinical implications, suggesting that parameter selection should be tailored to the phase of recovery, with acute protocols prioritizing neuroprotection via the cholinergic anti-inflammatory pathway and chronic protocols emphasizing noradrenergic-driven neuroplasticity for motor relearning [48,49]. For instance, in the case of neuroprotection, low-frequency stimulation reduced infarct size in a rat model of ischemic stroke [48].

## 4. The Role of Cholinergic System in VNS

The cholinergic system is a system of organized nerve cells that use the neurotransmitter, acetylcholine, in the transduction of action potentials [50,51] and, as such, it is a key modulatory pathway for both the central nervous system (CNS) and the peripheral nervous system [52]. The system comprises acetylcholine (ACh), cholinergic receptors (AChRs), choline acetyltransferase (ChAT) enzyme, and vesicular acetylcholine transporter (VAChT) (AChE) [50]. The Ach is synthesized from choline and acetyl-CoA with the aid of choline acetyltransferase (ChAT) enzyme. Following its synthesis, the Ach is then transported into vesicles and released into the synaptic cleft for it to bind with AchRs, which are majorly categorized into two, the nicotinic receptors (nAChRs) and the muscarinic receptors (mAchRs) [53].

The nAChRs are expressed in abundance in neurons of the entorhinal, temporal, and primary motor cortices and in the hippocampus and thalamus [50,54]. The mAChRs on the other hand are highly expressed in caudate nucleus and nucleus accumbens and, to some extent, in the somatosensory, primary motor, cerebellar and temporal cortices [50]. Expressions of nAChRs and mAChRs help decrease cell damage and death and enhance synaptic plasticity [52,53,54,55]. This is made possible because activation of brain nAChRs and mAChRs result in the release of various key neurotransmitters, including dopamine, serotonin, glutamate and γ-aminobutyric acid (GABA) [54,55,56]. These neurotransmitters also play a major role in modulation of learning and plasticity in the striatal, basal ganglia and hippocampal systems [57,58,59,60,61,62].

**Figure 2:**
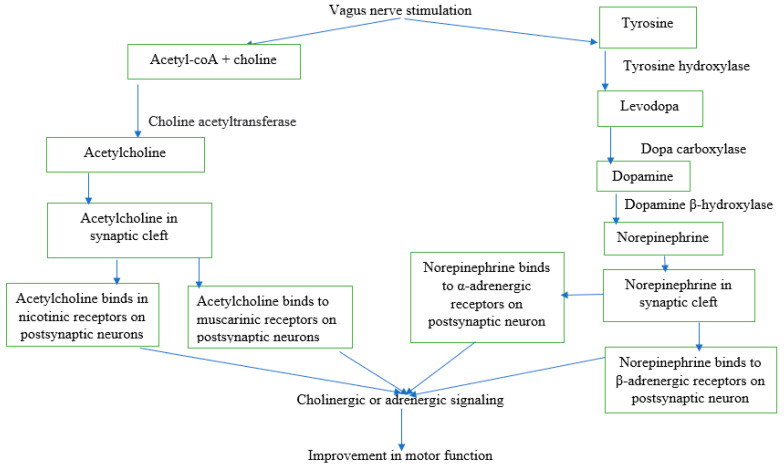
Roles of cholinergic and adrenergic systems in the recovery of motor function following stroke.

In the CNS, major cholinergic projections are the nucleus basalis of Meynert, supplying cholinergic projections throughout the cerebral cortex and hippocampus, and pedunculopontine nucleus pars compacta, projecting to the forebrain and subcortical structures such as thalamus and the striatal cholinergic neurons [50]. As such, the cholinergic system is involved in various physiological processes in the brain such as learning and memory, attention, sleep, arousal and regulation of homeostasis [50,63,64]. Of particular importance are the physiological processes, learning and memory, which play major roles in recovery of functions of the CNS [65,66,67,68]. The cholinergic system is involved in these two neurophysiological processes by modulating neural activity via acetylcholine receptors [69]. In recent studies, VNS improved perceptual learning and modulated the primary motor cortex [70,71,72]. (See Figure 2 for the putative role cholinergic system plays in VNS to improve motor function in patients with stroke.)

A critical mechanism through which the cholinergic system contributes to motor recovery following VNS is the facilitation of spike-timing-dependent plasticity (STDP) within the motor cortex. STDP is a cellular mechanism whereby the timing of pre- and postsynaptic action potentials determines whether synaptic strengthening or weakening occurs [73]. VNS, by delivering a burst of acetylcholine concurrently with motor training, provides a temporal window of enhanced excitability that reinforces Hebbian plasticity rules. Specifically, acetylcholine released from basal forebrain projections acts on muscarinic M1 receptors on pyramidal neurons, lowering the threshold for long-term potentiation (LTP) induction in synapses that are active during movement [74,75,76]. This mechanistic insight explains why pairing VNS with precisely timed motor events yields superior outcomes compared to unpaired stimulation, as demonstrated in both animal models and human trials [16,21]. Furthermore, the cholinergic system interacts synergistically with the noradrenergic system to modulate plasticity [77].

## 5. The Role of Adrenergic System in VNS

The adrenergic system comprises epinephrine, norepinephrine (NE), adrenergic receptors (ARs), tyrosine hydroxylase, dopa carboxylase and dopamine β-hydroxylase [52,77,78]. The NE is synthesized from tyrosine through a number of enzymatic reactions. At first, tyrosine hydroxylase (TH) catalyzes the reaction that sees tyrosine converted to L-DOPA, which is the main precursor for dopamine [77,78]. Thereafter, the L-DOPA is then converted to dopamine by the aid of catalytic activity of DOP dopa carboxylase [52,78]. Finally, the dopamine is converted to NE via catalytic activity dopamine-β-Hydroxylase (DBH) [52,77]. Consequently, NE is released from sympathetic nerve endings as a neurotransmitter, binds postsynaptically with the adrenergic receptors, α and β adrenergic receptors, and exerts its modulatory effects [52].

In the brain, the Locus Coeruleus (LC) is the main noradrenergic nucleus and the main site for the synthesis of norepinephrine [52]. The LC-NE system consists of neurons that project to different brain regions and supplies NE to the cortex, hippocampus, striatum, amygdala, cerebellum, basal forebrain, and the hypothalamus [79,80,81]. This is made possible because, in these regions of the brain, ARs are widely distributed [79,80,81]. Thus, the adrenergic system together with NE are involved in the process of memory and learning and play important roles in motor control, attention and cognitive functions [39,40,82,83]. Consequently, due to the numerous diffuse cortical connections of the vagus nerve, VNS can activate the adrenergic system to help recover motor function in conjunction with the cholinergic system. See Figure 2 for the putative role adrenergic system plays in VNS to improve motor function in patients with stroke.

The noradrenergic system’s contribution to VNS-mediated motor recovery extends beyond generalized arousal. Neuroimaging studies in humans have revealed that VNS-induced activation of the locus coeruleus leads to increased functional connectivity between the supplementary motor area (SMA) and the primary motor cortex (M1), a network that is frequently disrupted following stroke [84,85]. This enhanced connectivity correlates strongly with improvements in motor task performance, suggesting that norepinephrine acts to “re-couple” motor planning regions with execution regions [86,87]. At the molecular level, norepinephrine binding to β-adrenergic receptors on astrocytes triggers the release of neurotrophic factors such as brain-derived neurotrophic factor (BDNF), which supports the survival and differentiation of new neurons and the stabilization of new synapses in peri-infarct regions [88]. Notably, the effects of VNS on the adrenergic system appear to be lateralized, with greater noradrenergic activation in the ipsilesional hemisphere following unilateral stroke, indicating that VNS may help correct interhemispheric imbalances that characterize post-stroke motor dysfunction [89,90,91].

## 6. Evidence for the Effects of VNS from Animal Models and Human Studies

In mice, stimulation of the brain structures such as the basal forebrain results in upregulation of cholinergic neurotransmitter, the Ach [92,93]. Activation of the cholinergic system, specifically the α-7 nicotinic acetylcholine receptors, causes reduction in release of pro-inflammatory cytokines such as tumor necrosis α (TNF-α) and oxidative stress through the inflammatory reflex of the vagus nerve [42,43,45]. In addition, it increases the release of pro-angiogenic factors such as the interleukin-1 (IL-1) that helps to increase the expression of vascular endothelial growth factor (VEGF) [44].

In addition, reduction in pro-inflammatory cytokines will cause reduction in matrix-metalloproteinases (MMP), enzymes that cause disruption of the blood–brain barrier (BBB) [44,92,93]. Thus, collectively, activation of the cholinergic system will help reduce cerebral oedema and infarct volume [16,45]. Reducing inflammation, cerebral oedema and infarct volume can help reduce cellular, tissue and organ damage, and improve motor function [93]. Consequently, VNS has helped improved control of motor and control of skilled reaching in both animal stroke models and patients with stroke [15,16,17,18,19,20,21,22,92,94].

Furthermore, VNS is associated with reduction in ischemia-induced glutamate release and increase in Peroxisome Proliferator-Activator Receptor Gamma (PPAR-γ), Brain-derived Neurotrophic Factor (BDNF) and growth differentiation factor 11 (GDF-11) [95,96,97,98]. Reduced ischemia-induced glutamate release increases neuronal cells survival by reducing trigger spreading depolarizations (SDs) [99]. Similarly, increased levels of PPAR-γ, BDNF and GDF-11 promote angiogenesis and neurogenesis [95,96,98].

For the adrenergic system, NE modulates synaptic transmission and plasticity throughout the CNS by acting at G-protein coupled α and β adrenergic receptors (ARs) [84]. Thus, enhancement of level of NE, which aids with coupling of ipsilesional supplementary motor area (SMA) with primary motor cortex that correlates with improved motor performance, may purportedly be the mechanism through which VNS improves motor function in patients with stroke [84,100,101]. This is because improved functional connectivity of different brain areas involved in a particular brain function such as the motor function is one of the factors responsible for recovery after stroke [102,103]. Consequently, VNS paired with motor training enlarges the map representation of task-relevant musculature in the motor cortex through activation of cortical α2-ARs [85,104]. In addition, VNS increases the firing rates of noradrenergic neurons in the locus coeruleus [105].

The translation of these mechanistic findings into clinical practice has been supported by several pivotal human trials. The landmark VNS-REHAB trial, a randomized controlled trial involving 108 patients with moderate-to-severe upper limb impairment following ischemic stroke, demonstrated that VNS paired with intensive physical rehabilitation resulted in clinically meaningful improvements in motor function that were sustained for at least 12 months post-treatment [20,22]. Notably, the magnitude of improvement exceeded that observed with rehabilitation alone, providing high-level evidence for the adjunctive role of VNS. Mechanistically, this trial confirmed that the timing of stimulation relative to movement is critical; patients who received VNS synchronized with movement attempts showed significantly greater gains than those receiving non-contingent stimulation [22].

## 7. Implications for Rehabilitation

Since, aside from neurons, other organs in the body such as the skin, liver, eye, placenta and kidney are involved in the synthesis of Ach, VNS can be used to help raise the level of tissue and serum Ach [104]. This is because the vagus nerve extends and projects to diverse areas of the body, including the skin around the ear and the neck and most of the aforementioned organs. More importantly, stimulation of the skin may help reduce occurrence of adverse events. Thus, this argument supports the usability of the non-invasive method of VNS.

Similarly, determining the intensity of stimulation for optimal activation of the cholinergic and adrenergic systems is also important. At the moment, different studies used different stimulation parameters [15,16,17,18,19,20,21,22]. In addition, present protocols and results of NVS seem to suggest that it only performs an adjunctive role. Therefore, knowing the optimal intensity of stimulation that can help to independently improve neurophysiological outcomes and motor function can help prove this belief wrong. Furthermore, combining the stimulation with other interventions, as it is with most of the current protocols of VNS, can also help give the desired outcome. Combination of different interventions to help maximize favorable outcomes has been advocated [106,107,108].

Another factor worth considering is the characteristics of the patients. Probably genetic affinity to synthesis or release of cholinergic and adrenergic neurotransmitters and the receptors’ response to these neurotransmitters may differ between patients. Following constraint induced movement therapy, it has been argued that genetic affinity may play a role in the improvement [109]. Similarly, response to VNS and its ability to activate cholinergic and adrenergic systems may depend on time since stroke. Currently, in most of the VNS studies, the participants included were in chronic stage of stroke. However, response to treatment or rehabilitation depends on time since stroke, and the earlier the better [110,111,112,113]. In addition, in the majority of the current studies, the participants had an ischemic stroke, which is being regarded as the one that tends to have a more favorable outcome [114]. Thus, further research is needed to further determine how VNS modulates the nervous system following stroke.

Future clinical applications of VNS in stroke rehabilitation will likely focus more on personalized care and biomarker-driven protocols. For example, patients with higher baseline heart rate variability (HRV), a proxy for vagal tone, may have greater capacity for VNS-induced plasticity [115]. Thus, HRV will serve as a determinant of the intensity and/or type of VNS used for such a patient. Similarly, incorporating neurophysiological biomarkers such as electroencephalography (EEG)-derived measures of cortical excitability and functional near-infrared spectroscopy (fNIRS) measures of hemodynamic response could enable real-time titration of stimulation parameters to individual neurophysiological states [116,117].

Another future consideration is the combination of VNS with optogenetics. Optogenetics is a neuromodulatory technique that uses light to manipulate cells, typically neurons, which have been genetically manipulated to express light-sensitive opsins [118]. Recently, VNS was combined with optogenetics to prevent mice from renal injury [119].

## 8. Conclusions

In conclusion, VNS represents a promising neuromodulation strategy for enhancing motor recovery following stroke, with its therapeutic effects mediated primarily through coordinated activation of the cholinergic and adrenergic systems. The cholinergic pathway, acting through nicotinic and muscarinic receptors, reduces neuroinflammation, promotes neuroprotection, and gates cortical plasticity by lowering the threshold for long-term potentiation (LTP) induction. Concurrently, the noradrenergic system, driven by activation of the locus coeruleus, enhances functional connectivity between motor planning and execution regions, stabilizes newly formed synaptic connections, and supports neurotrophic signaling essential for long-term recovery. These seem to suggest that cholinergic and adrenergic systems are potential therapeutic targets for motor rehabilitation following stroke. Therefore, novel treatments and rehabilitation for patients with stroke should target them.

## Data Availability

No new data were created or analyzed in this study. Data sharing is not applicable to this article.
